# 

**Published:** 1836

**Authors:** 


### COLLABORATORS.

Romeyn Beck, M. D., Professor of the Institutes of Medicine and Medical Jurisprudence in the Medical College of the Western District of New York.

Samuel A. Cartwright, M. D., of Natchez, Mississippi.

A. Clapp, M. D., of New Albany, Indiana.

Thomas Fearn, M. D., of Huntsville, Alabama.

James C. Finley, M. D., of Lebanon, Illinois.

Samuel D. Gross, M. D., Professor of General and Pathological Anatomy Physiology, and Medical Jurisprudence in the Cincinnati College, Ohio.

John P. Harrison, M. D., Professor of Materia Medica in the Cincinnati College, Ohio.

John F. Henry, M. D., of Bloomington, Illinois.

S. P. Hildreth, M. D., of Marietta, Ohio.

Horatio G. Jameson, M. D., late Professor of Surgery in the Cincinnati College, Ohio.

Jared P. Kirtland, M. D., of Poland, Ohio

Charles Luzenburg, M. D., Professor of Theory and Practice of Medicine in the Medical College of Louisiana.

Joseph N. McDowell, M. D., Professor of Special and Surgical Anatomy in the Cincinnati College, Ohio.

Henry Miller, M. D., of Harrodsburg, Kentucky.

Joseph Ray, M. D., Professor of Natural Philosopy in the Woodward College, Cincinnati, Ohio.

John L. Riddell, A. M., Adjunct Professor of Chemistry and Lecturer on Botany in the Cincinnati College, Ohio.

Landon C. Rives, M. D., Professor of Obstetrics and the Diseases peculiar to Women and Children, in the Cincinnati College, Ohio.

James B. Rodgers, M. D., Professor of Chemistry and Pharmacy in the Cincinnati College, Ohio.

Gerard Troost, M. D., Professor of Chemistry and Mineralogy in the University of Nashville, Tennessee.

